# Exploring the influence of path environment factors on walking behavior in urban parks with configuration attribute control

**DOI:** 10.1371/journal.pone.0329278

**Published:** 2025-07-28

**Authors:** Wei Dong, Qi Kang, Gangkui Wang, Ruiqian Hou, Ping Liu

**Affiliations:** 1 Department of Environmental Design, School of Design, Xi’an Technological University, Xi’an, China; 2 Northwest Branch, Beijing Tsinghua Tongheng Urban Planning and Design Institute, Xi’an, China; 3 Beijing BLDJ Landscape Architecture Institute, Beijing, China; 4 Department of Product Design, School of Design, Xi’an Technological University, Xi’an, China; Changan University: Chang'an University, CHINA

## Abstract

The existing evidence regarding the influence of visual and physical environmental features of park pathways on walking behavior is limited and contentious, partly due to the lack of control over pathway configurational attributes and walking behavior measurement methods. This study addresses this gap by using space syntax and GPS to quantify the structural attributes and pedestrian counts within the pathways in Fengqing Park. Through stratified regression analysis, we examined the impact of environmental factors on walking behavior while controlling for pathway configurational attributes. The findings reveal that even after accounting for pathway configurational attributes, visual (R² = 0.223) and physical (R² = 0.173) environmental factors significantly affect walking behavior. Specifically, pathways with greater choice (β = 0.359, p < 0.01) and depth (β = 0.179, p < 0.05) are more favored. In terms of visual elements, landscape architecture (β = 0.143, p < 0.05) enhances walking behavior, while the presence of water bodies (β = –0.168, p < 0.01) negatively affects it. With respect to the physical environment, path width (β = 0.514, p < 0.01), length (β = 0.163, p < 0.05), and surface smoothness (β = 0.152, p < 0.05) play significant roles in influencing walking behavior. This study contributes to advancing our understanding of the impact of the environmental features of urban park pathways on walking behavior, reconciling research disparities in this domain, and providing guidance for the design and improvement of park pathways.

## Introduction

Physical activity (PA) is widely recognized as a critical factor in enhancing people’s quality of life. Inadequate levels of PA can lead to increased risks of physical illnesses [1–3]. Urban parks have been consistently emphasized in previous studies as important settings for promoting PAs [–6]. Research consistently indicates that individuals’ PA levels are influenced by park environments [7]. Scholars from diverse fields, such as public health, urban planning, and environmental design, have identified key park environment features that impact PAs across different scales. They advocate for interventions aimed at improving these crucial environmental attributes to increase visitors’ PA levels [4,8,9].

Previous research has revealed that environmental attributes at various levels within urban parks, including the neighborhood, overall park, and park activity zone levels, significantly influence visitors’ levels of physical activity (PA). At the neighborhood level, factors such as park proximity [10] and accessibility [11,12] have been shown to profoundly impact residents’ PA levels. On the broader scale of the overall park, features such as park size [12–14], paved area size [15], green coverage [16], and the availability of facilities [3,5,17] play crucial roles in affecting the PA levels of park users. Furthermore, at the park activity zone level, specific areas designated for activities [18,19], such as basketball courts, playgrounds, and pathways, present relatively high PA levels, with park pathways demonstrating the most significant associations with PA [20,21]. A particular study highlighted that the likelihood of engaging in physical activities in parks with paved pathways is more than seven times greater than that in parks lacking such amenities, underscoring the importance of further research on pathway environmental characteristics [20,21].

The evidence regarding the influence of park pathway environmental characteristics on physical activity (PA) is limited and contradictory. On the basis of existing research, pathway environments can be categorized into two main attributes: the visual environment and the physical environment. The physical environment of pathways includes pathway morphology and paving, pathway facilities, and the connectivity between the pathway and activity areas. However, studies, focussing primarily on the elderly population, have examined the associations of these two main attributes with walking behavior. In terms of the visual environment dimension, some scholars suggest that factors such as lower spatial enclosure [21], proximity to trees [21], and water bodies [22] encourage PA among visitors, although conflicting findings exist [23]. With respect to pathway morphology and paving in the physical environment, researchers have reported that longer lengths, moderate widths (3–3.9 m), and features such as plastic or paved paving encourage walking behavior [23]. Concerning pathway facilities, some studies indicate that the presence of amenities such as benches [3,23,24] and lighting fixtures [23] increases the likelihood of walking, whereas other research shows no statistical relationship between the number of benches and the probability of walking [25]. Regarding the connection between pathways and activity areas, one study suggested that pathways not linked to activity areas attract more visitors [23], which contradicts previous findings [26]. One potential reason for these disparities could be the lack of control over pathway structural attributes, which have been shown to significantly influence walking behavior [27,28]. Failure to account for these influential factors may introduce bias into walking environment research, resulting in distortions in research conclusions.

Another limitation of this study lies in the method used to measure walking behavior. Previous studies have relied primarily on on-site observations or activity diary surveys, both of which have inherent limitations. On-site observations are constrained by factors such as time, location, and environmental conditions and are susceptible to external disturbances, leading to potential errors or omissions in data recording [16]. Activity diary surveys, on the other hand, depend on participants’ cognitive and memory abilities. Given the complex and variable pathways in urban parks, respondents may struggle to accurately recall their movement trajectories, reducing data accuracy and reliability [29,30]. Additionally, recent studies have explored the use of WiFi sensing technology, which effectively captures the overall distribution and movement trends of visitors within a given area. However, this method is primarily suited for macrolevel analysis and lacks the precision required to track individual walking trajectories [31]. In contrast, GPS tracking technology offers significant advantages in walking behavior research. First, GPS tracking objectively and accurately records visitor trajectories, eliminating errors caused by memory recall limitations or observer bias inherent in traditional methods [29]. Second, GPS tracking minimizes participant interference. In dynamic environments, particularly in open spaces such as urban parks, visitor movement patterns are highly complex. Direct observational methods may unintentionally alter visitor behavior and require observers to have a high level of technical expertise. Even with map-based assistance, manual tracking is prone to omissions or inaccuracies [16]. GPS tracking, on the other hand, enables automated data collection without disrupting visitor activities, ensuring data completeness and accuracy [32]. Furthermore, GPS tracking offers substantial advantages in data processing. Its automated recording function significantly reduces the workload associated with manual data organization and minimizes secondary errors caused by human intervention [33]. Compared with traditional manual data collection methods, GPS tracking facilitates the rapid acquisition of large-scale, high-quality trajectory data, which can be efficiently processed and analyzed using specialized software, thereby increasing overall research efficiency and data reliability.

To address the identified knowledge gap, this study aims to conduct a quantitative investigation into the influence of park walking environments on walking behavior. Specifically, this paper examines the impact of specific park pathway environmental characteristics on walking behavior while controlling for pathway structural attributes. This study seeks to answer two main questions. First, with pathway structural attributes accounted for, does the visual environment of park pathways still significantly influence the number of walking visitors? If so, the study aims to identify which specific features are pivotal and to what extent they affect walking behavior. Second, with pathway structural attributes controlled for, does the physical environment of park pathways still significantly influence the number of pedestrians? Similarly, this study analyzes in detail which specific features are crucial in this context and the extent of their impact on walking behavior. By addressing these questions, the findings of this study will deepen our understanding of park environments conducive to promoting physical activity among citizens. Moreover, it offers empirically based recommendations for park designers and policymakers to enhance park environments and enhance citizens’ levels of physical activity.

## Methods

### Research Site

**Fig 1:**
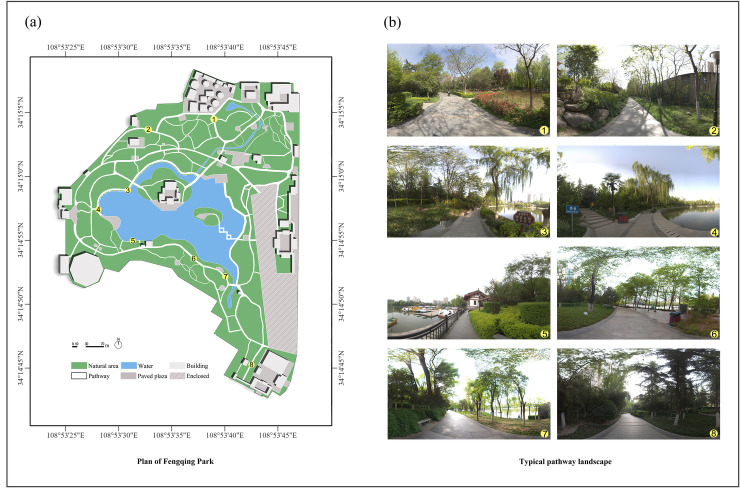
(a) Plan of Fengqing Park. (b) Park Scenes from Fengqing Park.

To ensure the representativeness of the study site, Fengqing Park was selected based on four main criteria: Fengqing Park was selected based on four criteria to ensure its representativeness: (1) Its location in a densely populated urban area with a high proportion of elderly residents. Xi’an, a major city in northwest China with a population of approximately 12.99 million as of 2022 [34], provides a typical urban context. Lianhu District, where the park is located, is one of the most prosperous and densely populated areas in the city and is characterized by a significant proportion of elderly residents. (2) Its excellent accessibility and high frequency of daily use by diverse user groups. Fengqing Park is situated in the southwest part of Lianhu District, surrounded by universities, commercial districts, museums, and residential communities, which makes it highly accessible and a popular destination for daily leisure and exercise. (3) Its diverse and typical environmental features. The park covers approximately 29 hectares and includes a wide range of pathway configurations and visual landscapes commonly found in large urban parks across China (Fig 1). These environmental characteristics offer valuable variation for analyzing walking behavior. (4) Its good maintenance and safety conditions. The park is well maintained, clean, and equipped with comprehensive safety measures, which ensured uninterrupted and reliable data collection throughout the study period. These attributes make Fengqing Park a typical and representative case for examining the relationship between park pathway environments and walking behavior, particularly among older adults in urban China.

In this study, pathways are defined as segments extending from one intersection to another, with no additional intersections along these routes. These pathways are considered units of analysis. A comprehensive survey of all pathways within Fengqing Park was conducted, excluding those with lengths less than 5 m, widths narrower than 0.4 m, and pathways leading to closed-off park areas. Pathways with lengths less than five meters are typically categorized as nodal pathways within the park, whereas those narrower than 0.4 m are often informal trails and thus were not included in the study scope. In total, 143 pathways met the criteria mentioned above.

### Dependent variable: Measurement of park visitor walking behavior

This study was reviewed and approved by the Academic Committee of the School of Design at Xi’an Technological University and complies with the relevant principles of the Declaration of Helsinki on human biological research. The collected questionnaires and trajectory data are anonymous and do not contain any personally identifiable information. The Academic Committee has also approved the use of oral informed consent from participants and assigned independent reviewers to oversee and witness the data collection process.

This study collects visitors’ social background information and activity trajectory data within the park to evaluate the impact of park segment environments on walking behavior. Data collection will be conducted between June 1, 2021, and June 1, 2024, at Fengqing Park in Xi’an.

The collection takes place in June during weather conditions suitable for outdoor activities, specifically between 8:00 AM and 11:00 AM and between 3:00 PM and 6:00 PM, as studies have shown that these are the peak visitation periods in parks. Researchers were stationed at the four entrances of the park. As the visitors entered, they explained the purpose of the study and the data collection process and then invited those whose primary activity was leisure physical activity to participate. Upon agreeing to participate, visitors provided oral informed consent under the supervision of reviewers appointed by the academic committee. In accordance with previous methods [35], researchers assisted participants in installing the “Two-Step Route” app, a widely used GPS outdoor recreation tracking tool in China, on their smartphones. Participants were reminded to add the app to their phone whitelist to ensure uninterrupted data recording. Upon completing their park activities, the participants returned to the park exits, where staff guided them in retrieving the GPX-format data files from their devices and asked them to complete a questionnaire. As a token of appreciation for their cooperation, small gifts were provided to the participants upon completion of the questionnaire. Through this data collection process, a total of 263 samples were obtained.

For data analysis, we initially imported the collected GPS trajectory data into ArcGIS software. We conducted preliminary processing by excluding samples with a duration of less than 3 min. We subsequently overlaid participants’ location trajectories onto the park road network layer and visually inspected them to exclude trajectories with significant data loss, substantial displacement of positioning points (more than 10 m from the road segment boundaries), and trajectories with distances shorter than 300 m. Ultimately, we obtained 218 valid samples, representing an 82.8% validity rate, with a total distance traveled of 364.836 kilometers.

**Fig 2:**
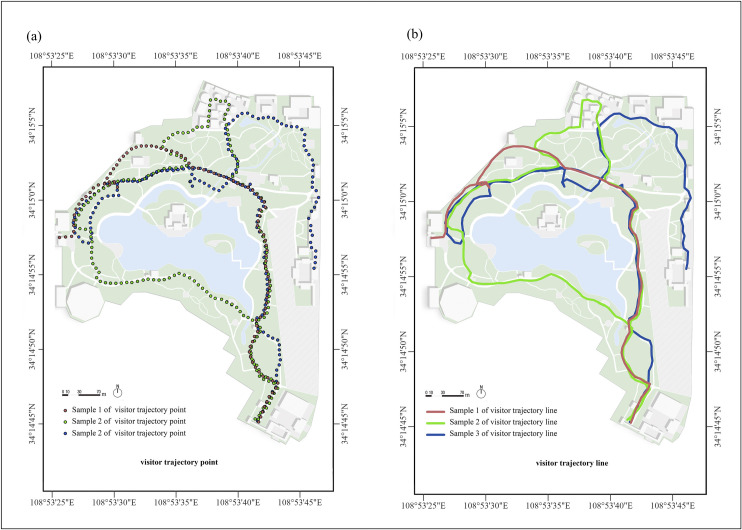
(a) Raw trajectory point samples of park visitors' walking behavior. (b) Conversion of trajectory points into trajectory lines.

To quantify the number of pedestrians on each pathway, we utilized the point-to-line tool in ArcGIS to convert the 218 location trajectories into vector line features (Fig 2). We subsequently overlaid these trajectory lines with the park road network boundary data and counted the trajectories passing through each park pathway as the dependent variable representing the number of pedestrians. Importantly, because the GPS positioning accuracy is within 10 m and considering that the path width is typically smaller than the error of the positioning sensor, some trajectories might extend beyond the pathway network. In light of this, we conducted visual inspections and included trajectories within a ten-meter range of the path boundaries in the total pedestrian count statistics. Additionally, to correct excessive deviations in a small number of positioning points caused by sporadic signal loss, visual adjustments were made on the basis of the spatial positions of preceding and succeeding points.

### Independent variables – Measurement of park pathway configurational attributes

We applied spatial syntax theory and its associated tool, DepthMap, to quantify the structural attributes of pathways in Fengqing Park. Spatial syntax theory provides a framework for describing architectural and urban spatial patterns via principles from graph theory. It abstracts the connections between spaces into connection graphs and conducts topological analysis to derive quantitative indicators of spatial organizational structures [36]. DepthMap, a spatial quantitative analysis and visualization tool based on spatial syntax theory, was utilized in this study. To obtain the structural attributes of the pathways in Fengqing Park, we followed a three-step process using the DepthMap axial model. First, spatial location data of pathways in Fengqing Park were extracted from the park’s master plan and Google Earth images. Based on the principle of accessibility, connection graphs between the 143 park pathways were drawn via AutoCAD. Second, the pathway connection graph was exported to a DXF file and imported into DepthMap software to transform it into an axial map of park pathways. Spatial syntax theory offers various measurement units, such as axial models and convex polygon models. The axial model summarizes each subspace in the spatial system as an axial line, which is used as the unit of measurement. In this study, which focused on the organizational structure of park roads, the axial model, with axial lines as the modeling unit, is deemed more suitable for characterizing park road attributes [37]. Third, we calculated four commonly used structural attribute variables for the 143 pathways in Fengqing Park: choice, connectivity, total depth, and integration, as detailed in Table 1. Finally, we applied color coding on the basis of the calculated values to visualize the pathways.

**Table 1:** Spatial syntactic variables description and computational formulas used in this study.

Variables	Definition	Formula	Explanation of the Formula	Literature Source
Choice	Choice indicates the number of times a segment is traversed along the shortest path between every pair of origins/destinations in the system	$\begin{array}{c} C_{i}=\sum_{j}\;\sum_{k}\;g_{jk}(i)/g_{jk}(j<k) \end{array}$	where $g_{jk}(i)$ is the number of shortest paths between lines j and k containing i,and $g_{jk}$ is the number of all shortest paths between j and k	[38]
Integration	The sum of the distances from one location to all other lines	$I_{i}=\frac{2\left(n\left(\log_{2}\left(\frac{n+2}{3}\right)-1\right)+1\right)/(n-1)(n-2)}{2\left(\left(\frac{\sum_{j=1}^{n}\;d_{ij}}{n-1}\right)-1\right)/(n-2)}$	Where n is the number of axial lines in the urban street area considered,$d_{ij}$ is the shortest distance (least number of steps) between two axial lines i and j.	[38,39]
Depth	The minimum number of spatial transitions required to reach other paths in a system	$\begin{array}{c} D_{i}\;=\sum_{j=1}^{n}\;d_{ij} \end{array}$	where $d_{ij}\;$is the shortest paths between lines i to j,	[40]
Connectivity	The number of lines directly connected to an line	${Con}_{i}=k$	where k is the number of lines that are connected to line i,i=1,2,3,…,n;n is the total number of lines in the system;$\;{Con}_{i}$ is the connectivity value of space i.	[41]

### Independent variables – Measurement of park pathway environment

The pedestrian-friendly environment within parks typically comprises pathway visual and physical attributes. Pathway physical attributes generally include pathway morphology and pavement, pathway amenities, and the connection of pathways with activity zones. The visual environmental variables along the path edges include the quantity of green vegetation species, the quantity of flower species, the density of large-canopy trees, lateral visibility, the degree of tree shade, the presence of water bodies, and architectural structures. The physical environmental variables primarily include pathway length, width, pavement smoothness, the presence of benches, light fixtures, signboards, and trash cans. The connectivity of pathways at both ends pertains to their connection with activity zones. These variables are derived from established park environmental audit tools or environmental features previously reported to significantly influence pedestrian behavior, as detailed in S1 Table. In June, we conducted an on-site examination of park pathway environmental features based on the definitions and procedures outlined in these audit tools. A trained researcher performed the observations, surveying each pathway twice. In cases of discrepancies between the two assessments, a third review was carried out until consistency was achieved.

### Analytical methods

In this study, we utilized SPSS Statistics 26 for data analysis, which comprises four main steps: descriptive statistics, collinearity testing, hierarchical regression construction, and analysis of variance (ANOVA) with post hoc multiple comparisons. Initially, a thorough descriptive statistical analysis was performed on the environmental variables of the pathways and pedestrian behaviors to establish sample characteristics. This procedure aimed to deepen the understanding of the data structure and set the foundation for subsequent analyses. Subsequently, collinearity among the independent variables was examined, and variables with variance inflation factor (VIF) values exceeding 10 were eliminated to ensure the validity of the regression model. This screening process improved the stability and interpretability of the model. In the third stage, the data were categorized into three levels based on the attributes of the independent variables and integrated into the regression model. By considering the structural attributes of the pathway, the influence of the physical and visual environments of the pathway on pedestrian behavior was evaluated. This hierarchical approach helps to uncover the distinct contributions of different environmental factors to pedestrian behavior in greater detail. Finally, ANOVA and post hoc multiple comparison methods were utilized to conduct further in-depth analysis of key independent variables significantly affecting pedestrian behavior on the pathways. This step facilitated a more comprehensive understanding of how environmental factors significantly influence pedestrian behavior. Through these processes, the mechanisms by which pathway environments impact pedestrian behavior were comprehensively and systematically elucidated, offering substantive guidance for future urban planning and design initiatives.

## Results

### Descriptive statistics

#### Walking behavior.

A total of 263 park visitors participated in this study, with 218 individuals (82.8%) providing valid GPS data paired with questionnaire responses; thus, these visitors were included in the analysis. The background information collected from the participants is detailed in Table 2. The gender distribution indicated that males comprised 59.17% of the participants, whereas females comprised 40.83%. In terms of age distribution, youth represented 6.97%, and middle-aged and elderly individuals represented 93.03%. Educational levels included junior high school or below (17.92%), high school/vocational school (43.4%), and bachelor’s/master’s/doctoral degree (2.83%). The occupations included national civil servants (30.19%), farmers (6.6%), professionals in commerce and service industries (6.13%), and other occupations (57.07%). The number of individuals in each pedestrian group was categorized as one person (58.69%), two persons (29.11%), or three or more persons (12.2%).

**Table 2:** Descriptive statistics of participant socio-demographic information.

Category	Type	Frequency	Percentage (%)
Gender	Male	129	59.17
	Female	89	40.83
Age	Youth	15	6.97
	Middle-aged and Elderly	200	93.03
Education Level	Junior high school or below	38	17.92
	High school/Technical secondary school	168	43.4
	Bachelor’s/Master’s/Doctoral	6	2.83
Occupation	Civil servant	64	30.19
	Farmer	14	6.6
	Business/Service sector employee	13	6.13
	Other occupations	121	57.07
Number of people in this trip	One person	125	58.69
	Two people	62	29.11
	Three people and above	26	12.20

Note: In this study, the integrity of tourists’ GPS tracks was used as the standard to judge the validity of the sample. In the survey questionnaire corresponding to the trajectories, some samples lacked socio-demographic attributes. Therefore, in the statistical results presented in Table 2, the sum of samples for certain attributes may not necessarily equal the total number of valid samples.

**Fig 3:**
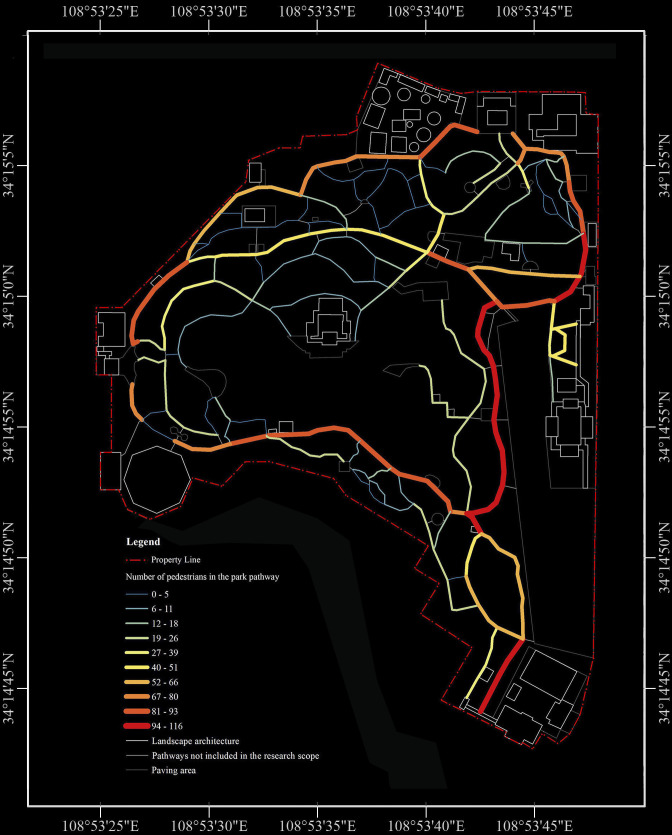
Walking visitor counts on various pathways within the park.

Pedestrian behavior was assessed at the pathway level. We tracked the walking counts of all participants on each pathway via GPS devices, which represented pedestrian behavior on each pathway. Across the 143 study pathways, the average number of pedestrians per pathway was 32.238 people (with a maximum of 116 people, minimum of 0 people, SD = 31.897, median = 20 people), as depicted in Fig 3.

#### Pathway configurational attributes.

**Fig 4:**
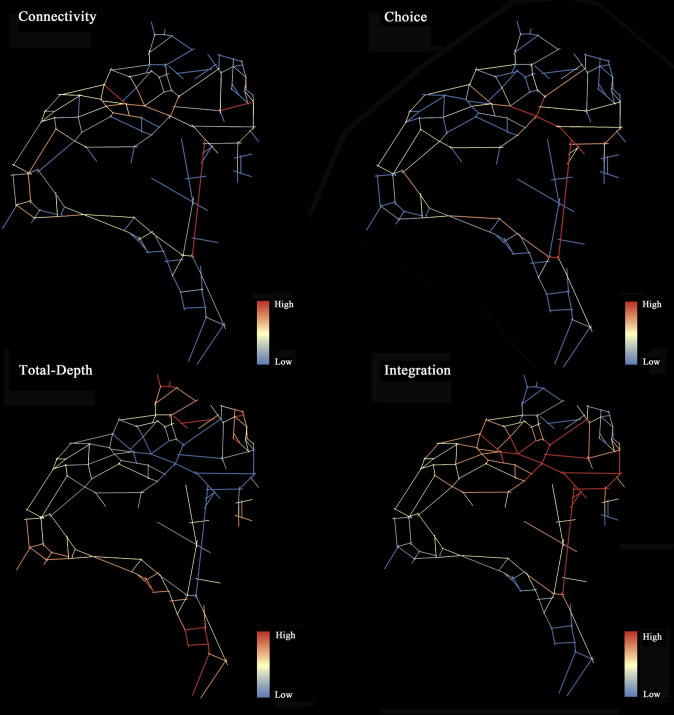
Visualization of the four structural attributes of park pathways.

Descriptive statistics for four measures of pathway configurational attributes are presented in Table 3. The visualization of these measures across pathways is illustrated in Fig 4. The average choice score for pathways is 1134.252 (with a maximum of 7217, a minimum of 0, a standard deviation of 1439.634, and a median of 597). The average connectivity score is 4.049 (with a maximum of 9, a minimum of 1, a standard deviation of 1.329, and a median of 4). The average integration score is 0.809 (with a maximum of 1.16, a minimum of 0.547, a standard deviation of 0.128, and a median of 0.799). The average total depth score is 1183.371 (with a maximum of 1637, a minimum of 640, a standard deviation of 164.158, and a median of 1174).

**Table 3:** Descriptive statistics of variables included in the analyses.

Variable Type	Variable		Mean	SD	Frequency	Percentage
Independent Variable – Pathway Configurational Attributes	Connectivity		4.049	1.329		
	Depth		1183.371	164.158		
	Choice		1134.252	1439.634		
	Integration		0.809	0.128		
Independent Variable – Pathway Visual Environment Attributes	Quantity of green vegetation species		6.406	2.686		
	Quantity of Flower Species		0.839	0.947		
	Large Canopy Tree Quantity Level					
		≤2			34	23.78
		3-4			53	37.06
		5-6			49	34.27
		≥7			7	4.90
	Lateral visibility					
		Low			20	13.99
		Moderately Low			34	23.78
		Moderate			41	28.67
		Moderately High			23	16.08
		High			25	17.48
	Degree of Tree Shade					
		<20%			7	4.90
		20%−39%			27	18.88
		40%−59%			38	26.57
		60%−79%			36	25.18
		80%−100%			35	24.48
	Presence of Water					
		No			110	76.92
		Yes			33	23.08
	Presence of Landscape Architecture	No			77	53.85
		Yes			66	46.15
Independent Variable – Pathway Visual Environment Attributes	Pathway Length (m)		55.455	42.537		
	Pathway Width (m)					
		<2m			27	18.88
		≥2m, < 3m			39	27.27
		≥3m, < 4m			18	12.59
		≥4m, < 5m			36	25.18
		≥5m			23	16.08
	Pavement Smoothness	Uneven			31	21.68
		Smooth			112	78.32
	Presence of benches	No			53	37.06
		Yes			90	62.94
	Presence of light fixtures					
		No			27	18.88
		Yes			116	81.12
	Presence of Signboards	No			57	39.86
		Yes			86	60.14
	Presence of Trash Cans					
		No			43	30.07
		Yes			100	69.93
	Pathway connection with activity zones	No			123	86.01
		Yes			20	13.99
Dependent Variable	Pedestrian Count		32.238	31.897		

#### Pathway environmental characteristics.

Table 3 presents descriptive statistics for the measurements of environmental attributes across 143 pathways. Concerning the visual environment of the pathways, the average quantities of green vegetation species and flower species are 6.406 and 0.839, respectively, with standard deviations of 2.686 and 0.947. Among all the pathways, 53 pathways included 3–4 large-canopy trees, accounting for the highest proportion (37.063%). In terms of lateral visibility, 41 pathways have moderate lateral visibility, representing the highest proportion at 28.67%. Pathways with over 40% tree shade coverage accounted for 76.22% of the total. Moreover, 23.08% of the pathways have visibility of water, whereas 46.15% offer views of park landscape architecture. In terms of the physical environment of the pathways, the average pathway length is 55.455 m, with the longest pathway measuring 332 m and the shortest measuring 8 m, with a standard deviation of 42.537. Among the pathway widths, 39 pathways were measured between 2 and 3 m, representing the highest proportion at 27.27%. Additionally, smooth pavement is present in 78.32% of the pathways. In terms of lighting fixtures, lights are available in 81.12% of the pathways, whereas public benches are found in 62.94%, signboards in 60.14%, and trash cans in 69.93%. Finally, 20 pathways (13.99%) are connected to park activity zones upon examination.

#### Inferential statistics.

We developed a three-tier regression model to explore the factors impacting pedestrian behavior (visitor count) in urban park pathways. We examined the influences of three aspects on pedestrian behavior in park pathways: pathway configuration, the visual environment, and the physical environment. After eliminating the Integration variable due to its VIF exceeding 10, as depicted in Table 4, the highest VIF value among the remaining independent variables in the three models is 2.926, indicating that there is no multicollinearity among them. The adjusted R-squared values for the three models are 0.277, 0.479, and 0.643, respectively, suggesting that the three sets of independent variables significantly affect pedestrian behavior on park pathways, with the pathway configuration being the most influential. All the models passed both the F test and the t-test, and the coefficients of the variables remained consistent across the three models, indicating satisfactory model fit and robustness.

**Table 4:** Regression analysis of pathway environment and walking behavior.

	Model 1			Model 2			Model 3		
	VIF	p	β	VIF	p	β	VIF	p	β
Constant	–	0.211	–	–	0.996	–	–	0.017*	–
Connectivity	1.471	0.073	−0.157	1.712	0.127	−0.122	1.807	0.048*	−0.135
Depth	1.852	0.005**	0.275	2.183	0.12	0.14	2.31	0.02*	0.179
Choice	1.858	0**	0.721	2.627	0**	0.569	2.926	0**	0.359
The quantity of green vegetation species				1.47	0**	0.271	1.902	0.714	0.025
Quantity of Flower Species				1.196	0.029*	−0.146	1.267	0.174	−0.077
Large Canopy Tree Quantity Level				1.19	0.291	0.07	1.246	0.215	0.07
Lateral Visibility				1.351	0.03*	−0.155	1.662	0.103	−0.106
Degree of Tree Shade				1.364	0.009**	−0.187	1.567	0.63	−0.03
Presence of Water				1.228	0.039*	−0.14	1.311	0.004**	−0.168
Presence of Landscape Architecture				1.12	0**	0.23	1.259	0.012*	0.143
Pathway Length							1.547	0.01*	0.163
Pathway Width							2.355	0**	0.514
Pavement Smoothness							1.938	0.031*	0.152
Presence of benches							1.348	0.444	−0.045
Presence of light fixtures							1.7	0.128	0.1
Presence of Signboards							1.5	0.31	−0.063
Presence of Trash Cans							1.731	0.074	−0.119
Pathway connection with activity zones							1.187	0.846	0.011
R^2^	0.293			0.515			0.688		
Adjusted R^2^	0.277			0.479			0.643		
F-value	F (3,139)=19.173,p = 0.000			F (10,132)=14.033,p = 0.000			F (18,124)=15.190,p = 0.000		
△R^2^	0.293			0.223			0.173		
△F-value	F (3,139)=19.173,p = 0.000			F (7,132)=8.660,p = 0.000			F (8,124)=8.579,p = 0.000		

Dependent Variable: Walking Behavior.

*p < 0.05, **p < 0.01.

In Model 1, both variables associated with pathway configurational attributes demonstrate a correlation with pedestrian behavior on park pathways. The path choice (β = 0.721, p = 0.000 < 0.01) and depth (β = 0.275, p = 0.005 < 0.01) of pathways significantly and positively affect pedestrian behavior on park pathways, with the influence of pathway choice being notably stronger than that of depth.

In Model 2, the pathway visual environment attributes were introduced. The outcomes of Model II indicate that the quantity of visible green vegetation species on both sides of the pathway (β = 0.271, p = 0.000 < 0.01) and the presence of landscape architecture (β = 0.230, p = 0.000 < 0.01) significantly positively influence the intensity of pathway usage. Conversely, lateral visibility, the quantity of flower species, the degree of tree shade, and the presence of water have negative impacts. Compared with Model 1, the introduction of new variables increased the adjusted R-squared value from 0.277 to 0.479, suggesting that visual landscape variables can account for 20.2% of the variance in the dependent variable.

In Model 3, which extends Model 2 by incorporating attributes of the physical environment, the results indicate that pathway width (β = 0.514, p = 0.000 < 0.01), pathway length (β = 0.163, p = 0.01 < 0.05), and smooth pavement (β = 0.152, p = 0.031 < 0.05) positively influence the utilization of park pathways. Compared with Model 2, the addition of new variables increased the adjusted R-square value from 0.479 to 0.643, suggesting that physical environmental attributes can explain 16.4% of the variance in the dependent variable.

## Discussion

This study employs a hierarchical regression model to examine the impact of park pathway environments on pedestrian behavior across three levels. In Model 1, the pathway configurational attributes demonstrate the highest explanatory power for pedestrian behavior (R² = 0.29), primarily reflecting the spatial topological structure of the park’s pathway network. This indicates that during the initial planning or renovation phases, space syntax techniques should be prioritized to optimize the pathway network layout, ensuring the structural rationality of the walking network and facilitating more efficient pedestrian circulation. In Model 2, the pathway visual environment attributes exhibit an explanatory power of R² = 0.22 for pedestrian behavior, involving the spatial arrangement of landscape elements along the pathway, such as visible vegetation, water features, and built structures. This suggests that, within the established pathway framework, attention should be given to optimizing the landscape configuration along walking routes, with a reasonable arrangement of vegetation, water features, and buildings to increase the attractiveness and comfort of the walking experience. Finally, in Model 3, the explanatory power of the pathway physical environment attributes is R² = 0.17, which mainly concerns pathway surface conditions and facility provisions. Although its impact is relatively small, it remains essential to focus on optimizing the path width, pavement smoothness, and related facilities to ensure pedestrian safety and convenience.

Specifically, consistent with prior research [42], the findings from Hierarchical Regression Model 1 indicate that park pathway structural attributes significantly affect pedestrian behavior, reaffirming its role as a crucial control layer in hierarchical regression. Notably, pedestrians prefer pathways with higher choice values. This observation is consistent with prior studies, where pathways with higher choice values tend to serve as more frequent connections between any two pathways in the network, making them more likely to be chosen by pedestrians [43–46]. Unlike conclusions drawn from previous research on urban streets, where higher depth values are associated with fewer pedestrians [40,47], our analysis reveals a positive influence of depth values on pathway pedestrian intensity. This deviation could arise from differing pedestrian motivations. Two studies, which are distinct from our study but related to urban streets, were also conducted in China and utilized DepthMap software for spatial network analysis, which is consistent with our research approach. However, these studies focused primarily on 84 road segments within urban block networks and small-town streets. In such settings, pedestrians are more likely to have utilitarian motivations, meaning that their walks are typically goal oriented, such as commuting to work, school, shopping centers, or other specific destinations. The main objective of such walks is to reach the destination, with less focus on the walking process itself. In these instances, time and distance are likely the primary factors influencing pathway selection, leading to a preference for pathways with lower depth values, indicating greater accessibility. On the other hand, in research concerning urban park pathways, pedestrians participate in activities such as leisurely strolls and fitness walks, emphasizing nonutilitarian motives such as recreation, entertainment, exercise, or simply enjoying their surroundings. In such cases, pedestrians may not have specific destinations in mind but rather enjoy the interactive experience with the surrounding pathway environment. As a result, park pedestrians may prioritize pathways that facilitate exercise and offer scenic enjoyment when making pathway choices.

The results from Model 2 reveal that six visual landscape features of park pathways are linked to pedestrian behavior, explaining 22.3% of the variance. These findings address Research Question 1, indicating that even with pathway structural attributes controlled, visual environmental cues still significantly influence pedestrian behavior. Specifically, a significant positive correlation is observed between the quantity of visible green plant species on either side of the pathway and pedestrian behavior, which aligns with prior research [14]. The Biophilia hypothesis suggests that biodiversity in natural environments enhances human satisfaction and well-being, as individuals exhibit greater curiosity and interest in diverse landscapes [48]. This increased curiosity and interest may prompt individuals to visit and explore such places more frequently. According to forest therapy theory, interacting with nature, particularly diverse plants, can reduce stress levels, alleviate anxiety, enhance mood, and increase attention [49]. As individuals stroll amid the diverse plant life surrounding park pathways, they may experience increased relaxation and contentment, leading them to be more inclined to walk. The study findings also suggest a positive influence of visible landscape architecture on pedestrian behavior. This could be attributed to the visual appeal of landscape architecture and its connection to cultural identity. The landscape architecture in Fengqing Park offers significant visual allure, enhancing pedestrians’ visual enjoyment and thus encouraging more walking along these pathways. Additionally, the park architecture follows traditional Chinese garden styles closely linked to Xi’an’s local culture and history. According to place attachment theory, landscape architecture reflecting local cultural characteristics may evoke emotional attachments among residents [50], fostering greater exploration and interaction among citizens and thereby promoting increased visits to these pathways.

Unlike previous studies [21,51,52], our results indicate that the degree of tree shade coverage has a negative effect on walking behavior in park pathways. Two prior empirical studies from China and an in-depth interview study from Australia suggested that tree shade helps lower the temperature under the canopy and enhances visitor comfort. The contradictory findings in our study may be attributed to visitor preferences and the specific characteristics of the pathway samples in Fengqing Park. First, primary park visitors are middle-aged and elderly individuals who typically prefer sunlight in outdoor settings to promote health and psychological well-being, particularly in densely populated urban areas. Second, in Fengqing Park, 76.22% of the pathways presented shade coverage exceeding 40%, indicating a relatively low proportion of pathways with minimal shade. These factors likely contribute to the higher pedestrian traffic observed on pathways with less shade. Notably, the literature suggests that the effect of tree canopy coverage on walking behavior may vary significantly in winter due to factors such as leaf shedding and changes in sunlight exposure, which makes these findings incomparable to the summer-based findings of this study [53].

We observed a significant negative impact of water presence on pathway usage, which, when combined with previous research findings, suggests the existence of complex underlying mechanisms. Existing studies consistently demonstrate that water bodies significantly increase park visitation. Empirical research from Utah [5], Minnesota [14], and Melbourne [21] has shown that waterfront landscapes effectively increase park appeal and increase visitor numbers. However, the relationship between visitor behavior within parks and water spaces requires further investigation. Case studies from Turkey [54] and Shanghai [55] revealed a significant negative correlation between waterfront areas and the duration and intensity of visitors’ physical activity. This finding aligns with a systematic review covering studies from China, the U.S., Austria, and other countries [2], confirming that water spaces may reduce the intensity of recreational physical activities. Nonetheless, it is worth noting that current research lacks a systematic exploration of the intrinsic relationship between waterfront pathways and walking behavior.

Focusing on the empirical data from this study, we found a negative correlation between water-adjacent pathways and pedestrian volume. We believe that this outcome may stem from three potential factors. First, waterfront landscapes exhibit a dual nature of attraction and activity intensity. While water spaces can attract visitors to pause and enjoy the scenery, such behaviors are often static and low-intensity (e.g., photography, sightseeing) rather than encouraging continuous, high-intensity walking. This reduces the frequency of pedestrian passages per unit of time. In contrast, non-waterfront paths, which do not encourage stopping, tend to facilitate more frequent pedestrian movement. Second, walking behavior in parks is typically dominated by middle-aged and elderly individuals. Our study sample also primarily consists of this demographic data (see Table 2). Owing to safety concerns and walking efficiency preferences, this group may avoid waterfront paths that require crossing bridges or lead to dead-end areas, such as island sections. Third, the data collection took place during the summer season. The seasonal characteristics of the waterfront environment, including high humidity, mosquito infestations, and water-related odors, may collectively reduce walking comfort, thereby further discouraging pedestrian activity. This seasonal environmental constraint warrants further validation in future studies. On the basis of the above analysis, we conclude that pathways designed to promote recreational physical activity in parks should limit the presence of water bodies. This would help prevent prolonged visitor stays from diminishing walking behavior and reducing overall activity intensity.

Second, regarding the impact of flower species diversity on pathway walking intensity, this study revealed a negative association, which differs from the findings of previous studies. For example, studies conducted in Pune, India [2], Beijing, China [23], Guangzhou, China [56], and Vancouver, Canada [26], have shown that flowers and their diversity effectively attract visitors and, to some extent, promote walking activity in parks. However, in this study, we observed a negative correlation between flower species diversity and walking intensity. Further analysis of Model 3 (after adding pathway physical environment attributes) revealed that the variance inflation factor (VIF) value for flower species diversity only slightly increased, indicating that no significant multicollinearity issue occurred in the model. However, its regression coefficient significantly decreased by 0.069 and lost statistical significance. Additionally, the model’s explanatory power (R²) increased to 0.688, suggesting that the inclusion of new variables enhanced the model’s overall explanatory capacity for walking intensity while weakening the independent influence of flower species diversity. This phenomenon indicates that the newly added physical environment variables may have greater explanatory power for walking behavior, dispersing the contribution of the flower species variable and causing it to lose significance. With respect to the relationship between flower species diversity and walking behavior, we speculate that more complex mechanisms and diverse statistical variables may be involved, which are not fully understood in the present study.

**Fig 5:**
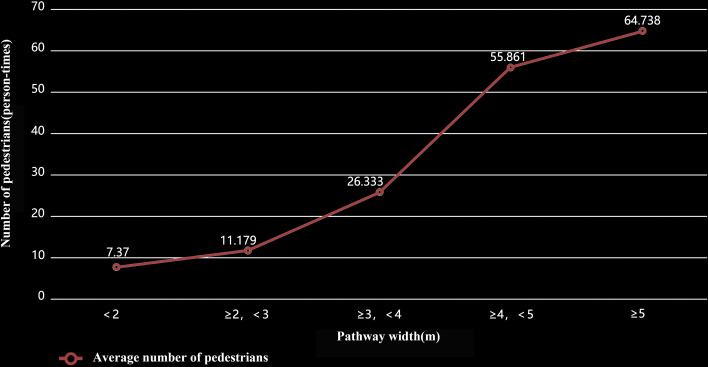
Average number of pedestrians on park pathways with different widths.

The findings of Model 3, which addresses Research Question 2, suggest that even after adjusting for path configurational attributes, the physical environment of pathways continues to significantly influence pedestrian behavior. Notably, pathway width emerges as a key factor, with a significant positive impact on pathway usage intensity (β = 0.514), surpassing other variables in significance. This observation resonates with previous research [57], indicating that wider pathways tend to attract more users. The wider space provided by these pathways likely facilitates various exercise and social activities during walking. To further explore this result, we conducted variance analysis and post hoc multiple comparisons (using the LSD algorithm) to examine differences in pedestrian numbers across pathways of various widths (see Table 5). Our analysis revealed that pathways wider than 5 m draw the greatest number of pedestrians, whereas the steepest growth curve in pedestrian numbers occurs in the 3–4 m width range, indicating significantly greater usage compared with other width ranges (see Fig 5). These findings highlight a positive association between increasing pathway width and a substantial increase in user numbers, particularly within the moderate width range, where pathway utilization exhibits the most pronounced trend.

**Table 5:** Analysis of variance and post hoc multiple comparisons (lsd) of pedestrian numbers across different road widths.

Variable Name	Pathway Width Measurement Value	Number of pathways	Average Pedestrian Count	SD	F	P
Pedestrian Count	<2m	27	7.37	7.458	41.708	0.000***
	≥2m, < 3m	39	11.179	13.286		
	≥3m, < 4m	18	26.333	23.778		
	≥4m, < 5m	36	55.861	26.212		
	≥5m	23	64.738	32.746		
	Total	143	32.238	31.897		
	**Pathway Width Measurement Value (I)**	**Pathway Width Measurement Value (J)**	**Average Pedestrian Count(I)**	**Average Pedestrian Count(J)**	**Difference (I-J)**	**P**
Pedestrian Count	<2m	≥2m, < 3m	7.37	11.179	−3.809	0.486
	<2m	≥3m, < 4m	7.37	26.333	−18.963	0.005***
	<2m	≥4m, < 5m	7.37	55.861	−48.491	0.000***
	<2m	≥5m	7.37	64.783	−57.412	0.000***
	≥2m, < 3m	≥3m, < 4m	11.179	26.333	−15.154	0.016***
	≥2m, < 3m	≥4m, < 5m	11.179	55.861	−44.682	0.000***
	≥2m, < 3m	≥5m	11.179	64.783	−53.603	0.000***
	≥3m, < 4m	≥4m, < 5m	26.333	55.861	−29.528	0.000***
	≥3m, < 4m	≥5m	26.333	64.783	−38.449	0.000***
	≥4m, < 5m	≥5m	55.861	64.783	−8.921	0.127

In line with previous studies [23], we noted a significant positive impact of pathway length on pedestrian behavior. This can be attributed to longer pathways offering more opportunities for physical activity and exercise. Walking, a form of aerobic exercise, has well-documented health benefits [57]. Longer pathways enable pedestrians to extend their walking distance, thus increasing their chances for exercise. Moreover, in park settings, longer paths typically mean fewer intersections and less pedestrian traffic along the way, reducing the need for pedestrians to navigate frequent obstacles and ensuring a smoother and more comfortable walking experience. Additionally, longer pathways suggest a more continuous landscape, providing walkers with a consistent environment. This continuity may increase pedestrians’ enjoyment, leading them to prefer longer paths for their walking activities.

The hierarchical regression analysis indicates that segments with smooth surfaces exhibit greater pedestrian activity, which is consistent with previous research findings [3]. To further examine the differential impact of surface smoothness on walking behavior, a variance analysis and post hoc multiple comparisons were conducted (Table 6). The average number of pedestrians on segments with smooth surfaces is 40.036, whereas on rough segments, it is only 4.065, resulting in a difference of –35.897 individuals. This difference is likely attributed to the provision of a more comfortable and safe walking environment with smooth surfaces. Pedestrians navigating smooth paths experience reduced concerns about tripping or falling, thereby minimizing the risk of injuries. Comfort and safety play vital roles in shaping the appeal of walking environments, especially among demographics such as elderly individuals, who prioritize safety. Moreover, on smooth surfaces, individuals who exercise typically maintain a swifter walking pace. The favorable surface conditions make walking more effortless, reducing resistance during movement and encouraging users to sustain higher speeds. This holds particular significance for park users selecting segments for exercise, as they tend to favor pathways conducive to aerobic activity that better meet their physical exercise needs.

**Table 6:** Analysis of Variance and Post Hoc Multiple Comparisons (LSD) for Pedestrian Numbers on Smooth and Uneven Pavements.

Variable Name	Pavement Type Measurement Value	Number of pathways	Average Pedestrian Count	SD	F	P
Pedestrian Count	Uneven	31	4.065	5.151	39.181	0.000***
	Smooth	112	40.036	31.897		
	Total	143	32.238	31.897		
	**Pavement Type Measurement Value (I)**	**Pavement Type Measurement Value (J)**	**Average Pedestrian Count(I)**	**Average Pedestrian Count (J)**	**Difference (I-J)**	**P**
Pedestrian Count	Uneven	Smooth	4.065	40.036	−35.971	0.000***

### Limitations and implications

The study has five primary limitations. First, the data collected are cross-sectional, limiting their ability to draw strong causal inferences. Second, the data were collected during the summer in Xi’an, Shaanxi Province, China, potentially restricting the generalizability of the findings across different regions or seasons. Third, certain potential confounding variables, such as the road gradient and congestion level, were not considered. Fourth, due to GPS positioning errors, some data points may have been incorrectly placed outside park road boundaries, leading to their exclusion from analysis. Fifth, inaccuracies in the park’s planning map and obstruction from tall trees in satellite images could result in errors in locating specific park sections. The study only collected data from a medium-sized comprehensive park in Xi’an, limiting the applicability of the findings to parks of different sizes.

Future studies should adopt more accurate methods for measuring pathway environments and walking behavior. Moreover, similar research should be conducted in parks across different regions, seasons, and scales to provide more empirical evidence. Finally, comparative studies of the results should be carried out to deepen the understanding of this research field. Despite these limitations, the findings of this study may still offer valuable implications for park design in other urban contexts. The observed hierarchical influence—where pathway configurational, visual, and physical attributes contribute progressively to walking behavior—suggests a generalizable framework that can inform design decisions in parks of different types and sizes. In large urban parks, optimizing the spatial structure using tools such as space syntax may be especially effective in guiding pedestrian flow. In contrast, in smaller or community-level parks, enhancing visual appeal and physical comfort may be more impactful. Additionally, adapting these strategies to local environmental conditions and user characteristics will be critical for their broader application.

## Conclusion

This study investigated the crucial environmental factors influencing walking behavior in urban parks. Research has shown that, even when accounting for pathway structural attributes, both visual (R² = 0.223) and physical (R² = 0.173) environments significantly impact walking behavior. Specifically, among pathway structural attributes (R² = 0.293), parks with higher values of choice (β = 0.359, p < 0.01) and depth (β = 0.179, p < 0.05) are preferred by visitors. With respect to visual landscapes, an abundance of green vegetation positively correlates with walking behavior, while the presence of landscape architecture (β = 0.143, p < 0.05) also has a positive impact, although the presence of water (β = −0.168, p < 0.01) has a negative influence. In terms of physical environments, path width (β = 0.514, p < 0.01), length (β = 0.163, p < 0.05), and pavement smoothness (β = 0.152, p < 0.05) significantly affect walking behavior. Wider pathways attract more users, whereas longer pathways offer increased opportunities for physical activity and exercise. Smooth pavement positively influences walking behavior by providing a comfortable and safe walking environment, which is particularly suitable for safety-sensitive groups such as elderly people. To our knowledge, this study is the first to explore the impact of park pathway environments on walking behavior while controlling for pathway structural attributes. The findings provide substantial recommendations for designers to improve and construct urban park pathways, contributing to the creation of more appealing urban park walking environments.

## Supporting information

S1 Table Operational definitions for pathway visual and physical attribute variables. (DOCX)

S2 File Supplementary data files. (ZIP)
