# Supplementary material for: Exploring the influence of path environment factors on walking behavior in urban parks with configuration attribute control
Source: PLoS One. 2025 Jul 28;20(7):e0329278. doi: 10.1371/journal.pone.0329278 (PMC12303266; doi:10.1371/journal.pone.0329278)
Supplement: S1 Table — (DOCX) [file pone.0329278.s001.docx]

**S1 Table. Operational Definitions for Pathway Visual and Physical Attribute Variables**

| **Dimension** | **Category** | **Variable** | **Type** | **Measurement** | **Reference Measurement Tool** | **Operational Definition** | **Justification** |
| --- | --- | --- | --- | --- | --- | --- | --- |
| Pathway Visual Environment Attributes | NA | Quantity of green vegetation species | Numerical | NA | Bedimo-Rung Assessment Tools-direct observation [1] | The observer moved slowly along the pathway, recording quantity of green vegetation species within a 5-meter range on one side of the pathway for every 10-meter segment. Upon reaching the end of the segment, the observer switched to the other side of the pathway and repeated the process. | The greenery in parks is a significant factor influencing the visitor experience [2]. Spending time in outdoor green spaces with diverse plants can improve physiological and psychological indicators [3]. |
|  |  | Quantity of Flower Species | Numerical | NA | Public Recreation Spaces Tool [4] | The observer moved slowly along the pathway, recording quantity of Flower Species within a 5-meter range on one side of the pathway for every 10-meter segment. Upon reaching the end of the segment, the observer switched to the other side of the pathway and repeated the process. | Color is important in influencing landscape preferences [2]. Older adults are concerned about their surroundings, including the smell and variety of flowers [5]. Flower-lined pathways can attract older adults [6]. |
|  |  | Large Canopy Tree Quantity Level | Categorical | a. ≤2; b. 3-4; c. 5-6; d. ≥7 | NA | The observer walked along the pathway, using a rangefinder to measure tree height (>20m), a laser distance meter to measure crown width (>8m), and Geographic Information System (GIS) data to verify canopy projection area (>45㎡) to determine the number of large-canopy trees within a 5-meter range on both sides of the pathway. Subsequently, based on the distribution of large-canopy trees on both sides of each surveyed segment, the pathways were classified into four levels: 2 trees or fewer, 3-4 trees, 5-6 trees, and 7 or more trees. | Older adults prefer parks with minimal disturbance and abundant trees and plants. High levels of physical activity are closely associated with variations in tree density [5]. |
|  |  | Lateral visibility | Categorical | a. Low; b. Moderate-Low; c. Moderate; d. Moderate-High; e. High | NA | Observers walked slowly along the pathway with a map in hand, assessing visual obstructions at eye level (1.6 meters). A laser ruler was used to measure the length of obstructed segments. Upon reaching the end of the segment, they switched to the opposite side and repeated the process. The proportion of obstructed segment length relative to the total segment length was then classified as follows: Low (≥90% of the segment is obstructed)、Moderate-Low (>60% obstruction)、Moderate (30%–60% obstruction)、Moderate-High (<30% obstruction)、High (≤10% obstruction) | Visual access can influence users' locomotion. Good visibility makes visitors feel safe and comfortable [5]. Visual access can influence users locomotion [7] |
|  |  | Degree of Tree Shade | Categorical | a. ＜20%  b. 20%-39%  c. 60%-79%  d. 80%-100% | Environmental Assessment of Public Recreation Spaces Tool [4] | The degree of tree shade is measured as the percentage of shade cast by trees (projected onto the ground) over the total area of the pathway segment. During sunny mornings between 10:00 - 11:00, observers walk along the pathway and visually estimate the amount of shade on the ground. | Shaded environments can make visitors feel safe and comfortable; high physical activity levels are closely associated with shade variation [5]. Shade conditions on park pathways are important for older adult use [8]. |
|  |  | Presence of water | Categorical | a. Not visible; b. Visible | Public Open Space Tool [9] | Observers walk along the pathway and check if water is visible | Water has positive impact on observers’ emotional state [10]. |
|  |  | Presence of landscape architecture | Categorical | a. Not visible; b. Visible | Community Park Audit Tool [11] | Observers walk along the pathway and check if landscape architecture is visible on both sides | Architecture can provide resting areas for older park visitors. Building height and enclosure levels can influence older adults' walking behavior [7]. |
| Pathway Visual Environment Attributes | Pathway Surface and Paving | Pathway length | Numerical | NA | Bedimo-Rung Assessment Tools-direct observation [2]. Environmental Assessment of Public Recreation Spaces Tool [4] | Measured in meters based on master plans and satellite imagery | Pathway length is positively correlated with the number of observed older adults [6]. |
|  |  | Pathway width | Categorical | a. < 2m, b. ≥2m， < 3m, c. ≥3m, < 4m, d. ≥4m， < 5m, e. ≥5m | Bedimo-Rung Assessment Tools-direct observation [2]. Environmental Assessment of Public Recreation Spaces Tool [4] | Measured in meters based on master plans and satellite imagery | Pathway width influences user experience [12]. |
|  |  | Pavement Smoothness | Categorical | a. Uneven (cobblestone, grass-paved); b. Smooth (bricks, plastic pathway) | Environmental Assessment of Public Recreation Spaces Tool [4] | NA | Trails with soft pavement are preferred [13, 14]. |
|  | Pathway Facilities Provision | Presence of  benches | Categorical | a. No benches on pathway sides; b. Benches on pathway sides | Community Park Audit Tool [11] | Observers walk along the pathway and check if benches are present on both sides | Benches can encourage more walking [15]; Seniors need resting opportunity [6]. |
|  |  | Presence of light fixtures | Categorical | a. No lights；  b. Have lights on sides | Environmental Assessment of Public Recreation Spaces Tool [4]. The Path Environment Audit Tool [16] | Observers walk along the pathway and check if light fixtures are present on both sides | Lights provide basic illumination and a sense of security to users, encouraging more visits [6]. |
|  |  | Presence of Signboards | Categorical | a. No signboards on pathway sides; b. Signboards on pathway sides | NA | Observers walk along the pathway and check if signboards are present on both sides | Signboards can provide guidance to pedestrians on pathways [8]. |
|  |  | Presence of Trash Cans | Categorical | a. No trash cans on pathway sides; b. Trash Cans on pathway sides | Environmental Assessment of Public Recreation Spaces Tool [4]. The Path Environment Audit Tool [16] | Observers walk along the pathway and check if trash cans are present on both sides | Trash cans serve pedestrians on pathways. |
|  | Other | Connection with  activity zones | Categorical | a. Not connected with activity zones; b. Connected with activity zones | Community Park Audit Tool [11] | Determined by the direct proximity of pathway sides to activity zones | Destinations lead to more walking [17]. Activity zones in parks are usually filled with events. If visitors want to participate in activities, these spaces can be considered attractive destinations [8]. |

References

1. Bedimo-Rung AL, Gustat J, Tompkins BJ, Rice J, Thomson J. Development of a direct observation instrument to measure environmental characteristics of parks for physical activity. J Phys Act Health. 2006;3(s1):S176-s89. doi: 10.1123/jpah.3.s1.s176. PubMed PMID: 28834515.

2. Donahue ML, Keeler BL, Wood SA, Fisher DM, Hamstead ZA, McPhearson T. Using social media to understand drivers of urban park visitation in the Twin Cities, MN. Landscape and Urban Planning. 2018;175:1-10. doi: /10.1016/j.landurbplan.2018.02.006.

3. Wang X, Rodiek S. Older adults’ preference for landscape features along urban park walkways in Nanjing, China. Int J Environ Res Public Health. 2019;16(20). Epub 20191010. doi: 10.3390/ijerph16203808. PubMed PMID: 31658651; PubMed Central PMCID: PMCPMC6843449.

4. Saelens BE, Frank LD, Auffrey C, Whitaker RC, Burdette HL, Colabianchi N. Measuring physical environments of parks and playgrounds: EAPRS instrument development and inter-rater reliability. J Phys Act Health. 2006;3(s1):S190-s207. doi: 10.1123/jpah.3.s1.s190. PubMed PMID: 28834520.

5. Lin M, Feng X. Relationship between visitor characteristics, physical activity levels and park environment in subtropical areas’ urban parks. Urban Forestry & Urban Greening. 2023;85:127958. doi: 10.1016/j.ufug.2023.127958.

6. Chudyk AM, Winters M, Moniruzzaman M, Ashe MC, Gould JS, McKay H. Destinations matter: The association between where older adults live and their travel behavior. J Transp Health. 2015;2(1):50-7. doi: 10.1016/j.jth.2014.09.008. PubMed PMID: 27104147; PubMed Central PMCID: PMCPMC4835227.

7. Borst HC, de Vries SI, Graham JMA, van Dongen JEF, Bakker I, Miedema HME. Influence of environmental street characteristics on walking route choice of elderly people. Journal of Environmental Psychology. 2009;29(4):477-84. doi: 10.1016/j.jenvp.2009.08.002.

8. Zhai Y, Baran PK. Urban park pathway design characteristics and senior walking behavior. Urban Forestry & Urban Greening. 2017;21:60-73. doi: 10.1016/j.ufug.2016.10.012.

9. Lange A, Giles-Corti B, Broomhall M. Quality of public open space tool (POST): Observers’ manual. Perth, Western Australia: School of Population Health, University of Western Australia; 2004.

10. Ulrich RS. Natural versus urban scenes: Some psychophysiological effects. Environment and Behavior. 1981;13(5):523-56. doi: 10.1177/0013916581135001.

11. Kaczynski AT, Stanis SA, Besenyi GM. Development and testing of a community stakeholder park audit tool. Am J Prev Med. 2012;42(3):242-9. doi: 10.1016/j.amepre.2011.10.018. PubMed PMID: 22341161.

12. Kaplan R, Kaplan S. The experience of nature: A psychological perspective. Cambridge: Cambridge University Press; 1989.

13. Kaplan R, Kaplan S, Ryan RL. With people in mind: Design and management of everyday nature. Landscape Journal. 1998;18(1):99-101.

14. Cohen DA, McKenzie TL, Sehgal A, Williamson S, Golinelli D, Lurie N. Contribution of public parks to physical activity. Am J Public Health. 2007;97(3):509-14. Epub 20070131. doi: 10.2105/ajph.2005.072447. PubMed PMID: 17267728; PubMed Central PMCID: PMCPMC1805017.

15. Lu Z. Investigating walking environments in and around assisted living facilities: A facility visit study. HERD: Health Environments Research & Design Journal. 2010;3(4):58-74. doi: 10.1177/193758671000300406.

16. Troped PJ, Cromley EK, Fragala MS, Melly SJ, Hasbrouck HH, Gortmaker SL, et al. Development and reliability and validity testing of an audit tool for trail/path characteristics: the Path Environment Audit Tool (PEAT). J Phys Act Health. 2006;3(s1):S158-s75. doi: 10.1123/jpah.3.s1.s158. PubMed PMID: 28834518.

17. GoliČNik B. Parks and their users. Urbani Izziv. 2008;19(2):133-9.
